# The clinical efficacy of collagen dressing on chronic wounds: A meta-analysis of 11 randomized controlled trials

**DOI:** 10.3389/fsurg.2022.978407

**Published:** 2022-08-31

**Authors:** Hongxin Shu, Zhiyu Xia, Xuan Qin, Xiaowei Wang, Weihang Lu, Qingyu Luo, Zhenxiong Zhang, Xiaowei Xiong

**Affiliations:** ^1^ Department of Vascular Surgery, The First Hospital of Nanchang, Nanchang, China; ^2^ Second Clinical Medical College, Nanchang University Medical School, Nanchang, China; ^3^ Vascular and Endovascular Surgery, the PLA General Hospital, Beijing, China

**Keywords:** collagen dressing, chronic wound, literature review, meta-analysis, trial sequential analysis, wound healing

## Abstract

**Objective:**

This study aims to evaluate the clinical efficacy of collagen dressing for patients with chronic wounds.

**Materials and methods:**

Relevant randomized controlled trials were searched from the databases such as PubMed, EMBASE, and the Cochrane library as of January 2022. For dichotomous outcomes and continuous outcomes, risk ratio and mean difference were calculated, respectively. Subgroup analysis was performed according to the type of chronic ulcer and follow-up. In addition, trial sequential analysis (TSA) was performed to further verify the results. Jadad score was used to assess the quality of trials. The Grading of Recommendations Assessment, Development, and Evaluation (GRADE) was utilized to assess the level of evidence for outcomes.

**Results:**

In 11 studies, a total of 961 patients of whom 485 were in the collagen group. Compared with standard of care (SOC) alone, the group that added an extra collagen dressing achieved a higher wound healing rate (Risk Ratio = 1.53; 95% CI, 1.33–1.77). The collagen group also showed a higher healing velocity than the SOC group (Mean Difference, 2.69; 95% CI, 0.87–4.51). In addition, the adverse events related to dressing between the two groups were similar (Risk Ratio = 0.67; 95% CI, 0.44–1.01).

**Conclusion:**

Collagen dressing increases the wound healing rate and may be an effective and safe treatment for chronic wound management. However, more extensive research shall be conducted to substantiate these results.

**Systematic review registration:**

https://www.crd.york.ac.uk/PROSPERO/display_record.php?RecordID=245728, identifier: CRD42021245728.

## Introduction

Skin injuries can be divided into acute and chronic wounds, according to the required time of healing. According to the definition, chronic wounds generally are wounds that cannot heal in 6 weeks (1). Chronic wound management was a challenging health issue. The most common typical chronic ulcers seen in lower limbs are venous/arterial leg ulcers and diabetic foot ulcers (DFUs) (2). Affected by a variety of factors, chronic wounds are highly lethal, with diabetic foot even higher than many cancer cases. At the same time, they have a massive impact on the quality of life, which is also an important reason why more attention should be paid. Furthermore, chronic wounds also have imposed a huge financial burden. In the US alone, more than 6 million patients suffer from chronic trauma annually, and it is estimated that the cost of the healthcare system is ranges from $20 to $25 billion (3).

Collagen is the most abundant and widely distributed functional protein in the body and contributes to wound healing. In the hemorrhagic coagulation phase, collagen has the role of hemostat due to three-dimensional scaffolds. After interacting with fibronectin and growth factors, collagen stimulates the chemotaxis of monocytes and fibroblasts, and then granulation tissue is produced (4). As a potential treatment, collagen dressings are applied in many fields such as chronic wounds, and reconstructive, separately or together with other materials (5). Meanwhile, Tiago et al. also demonstrated that collagen was the most efficient treatment for skin wounds (6). Therefore, collagen is in a dominant position in wound healing. For example, it can improve the ability of wound macrophages, accelerate epithelial cell formation, and promote angiogenesis (7–9).

At present, the role of collagen in wound healing is becoming more widely recognized. However, the effect of collagen dressing on chronic wounds was inconclusive (10). The goal of this article was to summarize the current evidence to evaluate whether superior outcomes can be obtained by adding extra collagen dressing to chronic wounds compared with standard wound care alone.

## Methods

The study protocol was prospectively registered at PROSPERO (registration no.: CRD42021245728) (https://www.crd.york.ac.uk/PROSPERO/display_record.php?RecordID=245728) and followed Preferred Reporting Items for Systematic Reviews and Meta-analysis (PRISMA) guidance (11).

### Search

The articles were systematically reviewed by searching in the PubMed, EMBASE, and the Cochrane library databases as of January 2022, which compared collagen dressing and standard of care with standard of care alone for chronic wounds. Additional references from eligible studies were systematically searched to determine any supplemental publications. Taking the PubMed database as an example, the keywords of search strategy are as follows: “collagen”, “dressing”, “chronic”, “wound”, “diabetic foot ulcer”, and “venous leg ulcers (VLCs)”.

### Eligibility

The inclusion criteria were as follows: (1) studies [published randomized controlled trials (RCTs)]; (2) participants (adult patients with typical chronic ulcers, such as VLUs, pressure ulcers, and DFUs); and (3) interventions (collagen-containing dressing is used in the experimental group, while conventional dressing, such as saline-moistened dressing, is used in the control group). The exclusion criteria were as follows: (1) studies that did not include original data; (2) studies that the type of article was review articles, editorials or conference abstracts, letters to the editor, and preclinical studies; (3) studies that did not report on previously defined outcomes; and (4) non-English text.

### Outcome parameters and definition

The primary outcome was wound healing rate because this was the most consistently reported outcome. The formula used to calculate wound healing rate is the number of patients who achieved wound healing during follow-up/the total number of observed patients. Wound healing was defined as complete closure as 100% re-epithelialization of the wound. Healing velocity, recurrence of ulceration, and adverse events (related to dressing) were the second outcomes. Healing velocity in follow-up duration was defined as the percentage of wound area per week that was reduced. Adverse events included amputations, skin sensitization, pain, discomfort, immunological rejection, infections, and off-odor et al.

### Data collection and extraction

Two independent researchers (HS, ZX) searched the database according to the search strategy designed previously and screened the retrieved studies as per the inclusion and exclusion criteria, and recorded the reasons for exclusion. If a dispute arose between the two researchers, it would be resolved by a panel discussion. Data extracted included the authors of the studies, country, year of publication, study design, population baseline, intervention, wound duration, wound area, and wound type.

### Critical appraisal

Based on Cochrane Collaboration's tool, the risk of bias 2.0 (RoB 2.0) was assessed by two researchers (HS, ZX) independently (12), and any dispute was resolved by a group discussion. To describe the quality of trials, the Jadad score was used, which was composed of randomization appropriateness, blinded outcome assessment, and a complete description of loss to follow-up (13). Grading of Recommendations Assessment, Development, and Evaluation (GRADE) was used to evaluate the overall level of evidence for each endpoint (14).

### Data synthesis

Data synthesis was performed by using STATA 17.0 software (StataCorp, College Station, TX). *I*^2^ statistical and *Chi*-square tests were used to calculate heterogeneity, and it was defined as high heterogeneity when *I*^2^ was greater than 50% and if it is less than 50% it was defined as low heterogeneity. In case of high heterogeneity, a random-effect model was used for the pooled results; otherwise, a fixed-effect model was performed on the pooled data. Forest plots were conducted to visualize the effect. It was defined that *P* < 0.05 was considered statistically significant. For dichotomous outcomes and continuous outcomes, the risk ratio (RR) and mean difference (MD) were calculated, respectively. To evaluate the stability of the meta-analysis results, sensitivity analysis was performed by removing one study from the analysis each time. Egger's and Begg’s tests were performed to further probe publication bias (15, 16). To find the source bias of data, a subgroup analysis of relevant studies was performed according to the types of wounds and the time of follow-up.

### Trial sequential analysis

In the present study, TSA v0.9.5.10 Beta Software was used to perform the trial sequential analysis (TSA). The required information size (RIS) is evaluated by setting relative risk reduction = 20%, the first type of error *α* = 0.05, and power = 80%. If the cumulative *Z*-curve crosses the RIS threshold, it means that the sample size of the accumulated evidence is sufficient. However, if the cumulative *Z*-curve does not cross the RIS threshold, it means that the sample size is not sufficient. Moreover, the result shall be confirmed by more studies. The TSA boundary is set based on the RIS. When the cumulative *Z*-curve crosses the TSA boundary, the conclusions are considered statistically significant.

## Results

### Literature search and selection

**Figure 1:**
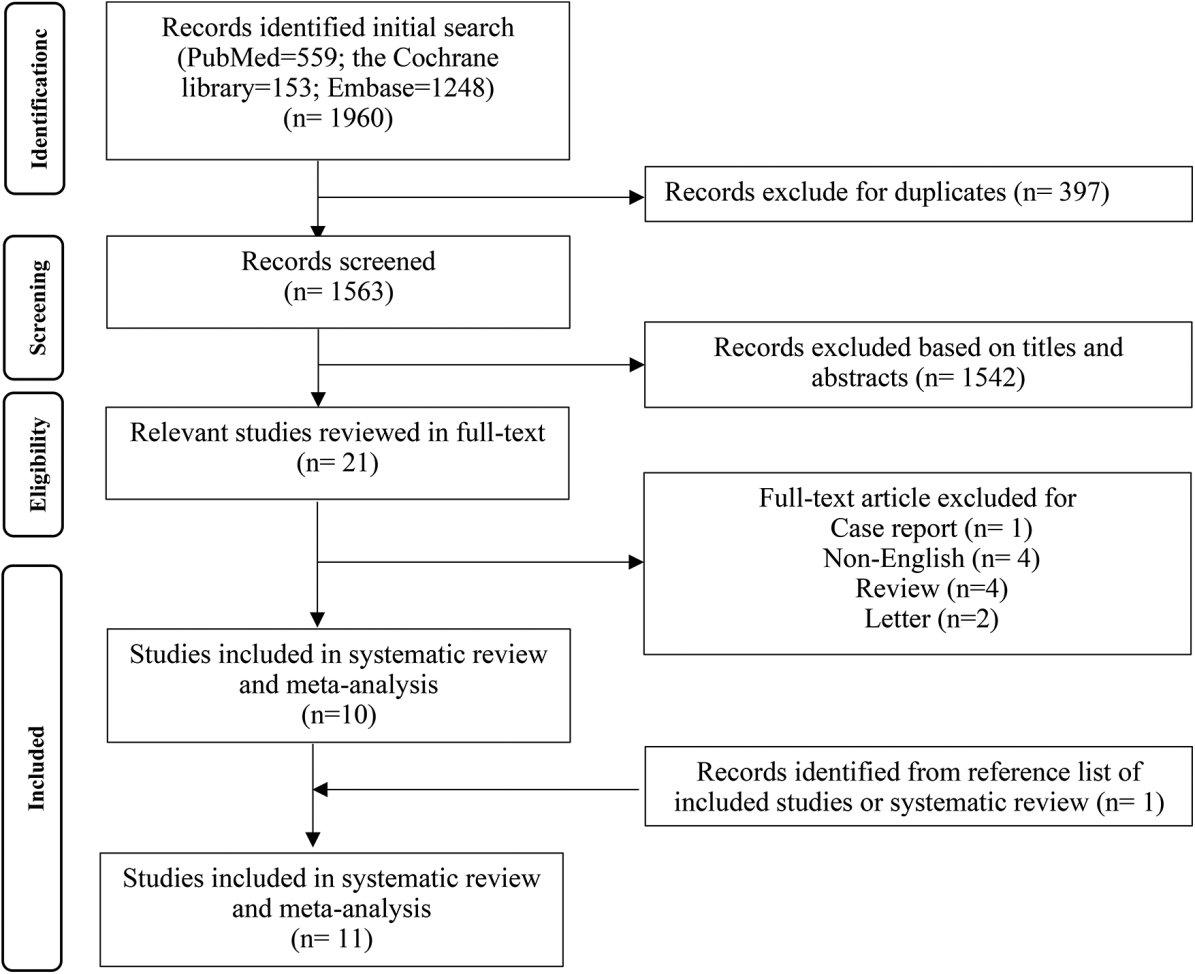
Flow diagram depicting the search strategy and study selection process.

After searching according to PICOS principles, 1,960 studies were initially retrieved, of which 21 were defined as potentially eligible. Among these 21 studies, one study was excluded for case report, four studies were excluded for non-English, four studies were excluded for review, and two studies were excluded for letter. The literature search and selection processes are described in Figure 1. In the end, 11 original studies were included in the current meta-analysis.

### Study characteristics

**Figure 2:**
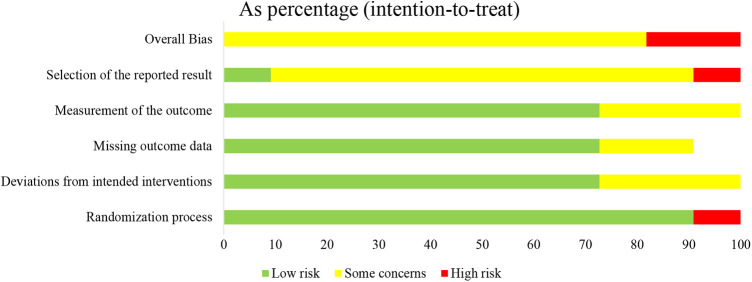
Methodologic quality diagram showing review authors’ judgments about each methodologic quality item presented as percentages across all included studies.

**Figure 3:**
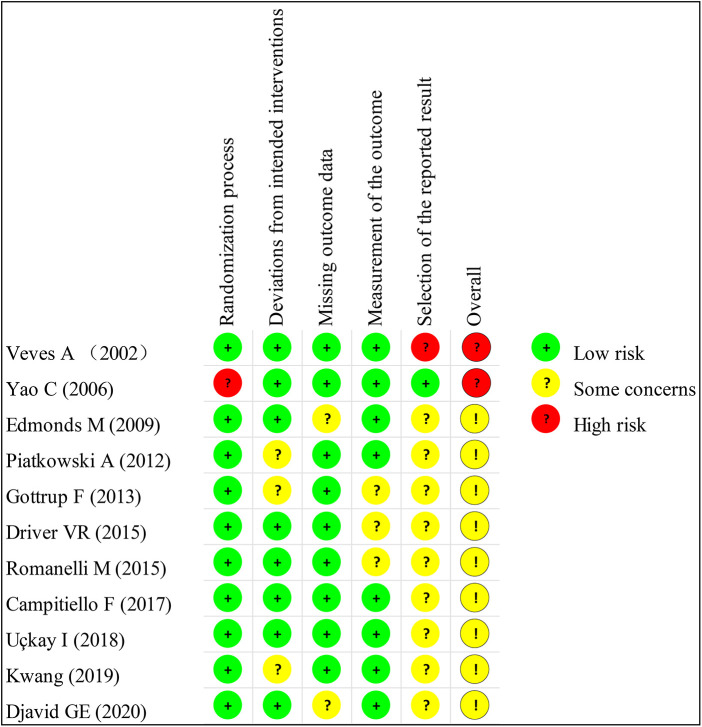
Methodologic quality summary showing review authors’ judgments about each methodologic quality item for each included study.

The basic characteristics of the included studies are described in Table 1, and the wound characteristics are described in Table 2 (17–27). A total of 961 patients, of whom 485 were in the collagen group, were enrolled in this study. Patients in the collagen group received collagen dressing and standard of care (SOC), while the control group underwent SOC alone. All these patients suffered from at least one chronic wound. Figures 2 and 3 show the risk of bias in the included studies. The results of the Cochrane RoB 2.0 tool indicated that two studies were considered high risk, and nine studies were considered moderate risk.

**Table 1:** Description of the studies in the meta-analysis.

First author	Year	Country	Sample (Collagen/Control)	Male, *n* (%)	Age (Mean)		Follow-up (Week)	Wound type	Jadad score
					Collagen	SOC			
Veves A	2002	United States	138/138	203 (73.6)	58	59	12	DFU	4
Yao C	2006	China	30/28	28 (48.3)	30	29	3	Traumatic	4
Edmonds M	2009	British	33/39	62 (86.1)	56	61	24	PU	3
Piatkowski A	2012	Germany	5/5	7 (70.0)	67	63	3	DFU	3
Gottrup F	2013	British	24/15	35 (89.7)	63	57	14	DFU	4
Driver VR	2015	United States	154/153	232 (75.6)	56	57	16	DFU	4
Romanelli M	2015	United States	20/20	12 (30.0)	68	65	12	VLU	3
Campitiello F	2017	Italy	23/23	28 (60.9)	64	62	6	DFU	4
Uçkay I	2018	Switzerland	11/11	14 (63.6)	69^a^	73^a^	1 month	DFU	4
Kwang	2019	Korea	17/13	23 (76.7)	63	53	12	DFU	4
Djavid GE	2020	Iran	30/31	40 (65.6)	54	57	24	DFU	3

DFU, diabetic foot ulcers; PU, pressure ulcers; VLU, venous leg ulcers; SOC, standard of care.

^a^ Data were described as median.

**Table 2:** Characteristics of the dressings and ulcers.

First author	Wound type	Ulcers area, mean (±SD), cm^2^		Ulcer duration time, mean (±SD)		Dressing	
		Collagen	Control	Collagen	Control	Collagen	Control
Veves A	DFU	2.5	3.1	3 months^a^	3 months^a^	Promogram	Moistened gauze
Yao C	Traumatic	24.7 ± 8.0	23.8 ± 7.2	19.07 ± 7.97	18.5 ± 7.69	rbFGF/ACS	Sterile gauze
Edmonds M	PU	3.0 ± 2.1	3.0 ± 2.1	1.1 years	1.2 years	Apligraf	Saline-moistened dressing
Piatkowski A	DFU	NR	NR	NR	NR	Collagen foam dressing	Foam dressing
Gottrup F	DFU	2.1 ± 3.1	4.4 ± 6.3	12.9 ± 13.0 months	16.9 ± 36.6 months	Collagen/ORC/silver	Standard treatment
Driver VR	DFU	3.5 ± 2.5	3.6 ± 2.7	308 ± 491 days	303 ± 418 days	Integra Dermal Regeneration Template	Sodium chloride gel
Romanelli M	VLU	26 ± 4	24 ± 5	24 ± 6 weeks	20 ± 4 weeks	Collagen membrane as a primary dressing	Alginate pad
Campitiello F	DFU	NR	NR	38.56 ± 12.61 weeks	39.50 ± 9.90 weeks	Integra Flowable Wound Matrix	Wet dressing
Uçkay I	DFU	NR	NR	NR	NR	Topical gentamicin–collagen sponge	Saline dressing
Kwang	DFU	5.0 ± 6.6	4.5 ± 6.6	17.4 ± 9.0 weeks	12.7 ± 6.7 weeks	100% porcine type I collagen dressing	Foam dressing
Djavid GE	DFU	3.1 ± 2.5	3.5 ± 4.2	NR	NR	Chitosan/collagen composite hydrogel materials	Saline-moistened gauze

DFU, diabetic foot ulcers; PU, pressure ulcers; VLU, venous leg ulcers; NR, not reported; Promogram, a collagen, oxidized regenerated cellulose dressing; rbFGF/ACS, recombinant basic fibroblast growth factor loaded on a kind of absorbable collagen sponge; ORC, oxidized regenerated cellulose.

^a^ Data were described as median.

### Pooled results

#### Wound healing rate

**Figure 4:**
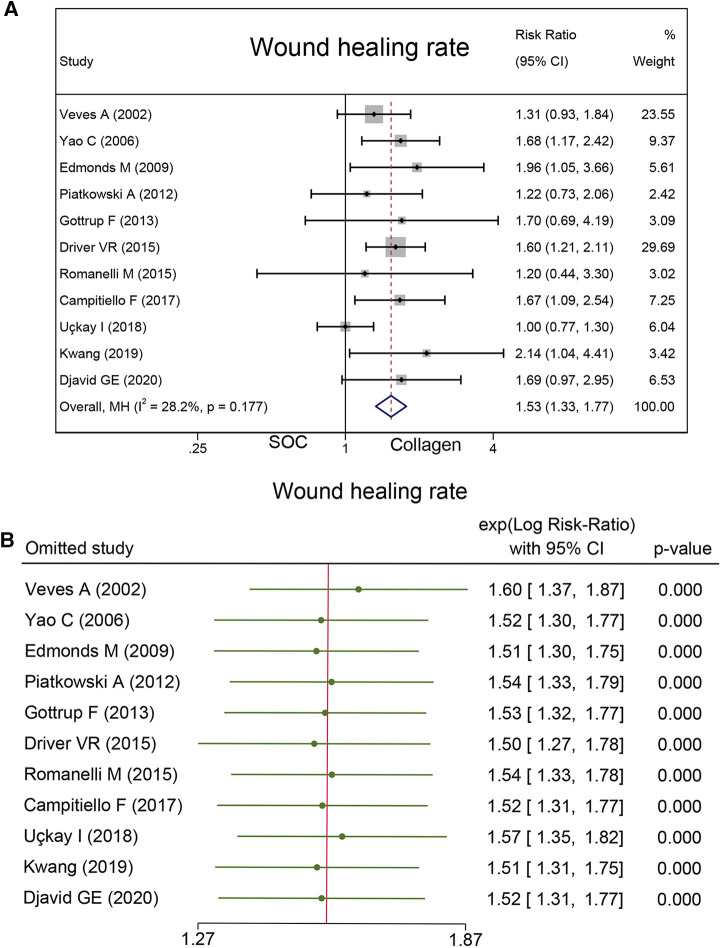
Wound healing rate: (**A**) the pooled result; (**B**) sensitivity analysis.

A total of 961 cases reported wound healing rate. In this endpoint, 53.4% (259/485) wounds in the collagen group and 34.50% (164/476) wounds in the SOC group achieved complete healing. Statistical analysis indicated that the wound healing rate in the collagen group is significantly higher than that in the control group (RR = 1.53; 95% CI, 1.33–1.77) (Figure 4A). No substantial heterogeneity was found as estimated by using the *I*^2^ statistic (*I*^2^ = 28.2%). No significant publication biases were found by Egger's (*P* = 0.14) and Begg's tests (*P* = 0.64). As Table 3 shows the subgroup analysis that was conducted based on follow-up and wound type. The source of heterogeneity may be explained by those factors.

**Table 3:** Subgroup analysis for wound healing rate.

Subgroup	No. of studies	No. of patients	RR (95% CI)	*I*^2^ (%)
Follow-up				
3-week	2	68	1.58 (1.16, 2.15)	3.32
4-week	1	22	1.00 (0.77, 1.30)	NA
6-week	1	46	1.67 (1.09, 2.54)	NA
12-week	5	725	1.48 (1.13, 1.94)	23.32
14-week	1	39	1.70 (0.69, 4.19)	NA
16-week	1	307	1.60 (1.21, 2.11)	NA
24-week	1	61	1.69 (0.97, 2.95)	NA
Wound type				
DFU	8	853	1.54 (1.31, 1.81)	45.39
PU	1	10	1.22 (0.73, 2.06)	NA
Traumatic	1	58	1.68 (1.17, 2.42)	NA
VLU	1	40	1.20 (0.44, 3.30)	NA

NA, not available.

#### Healing velocity

**Figure 5:**
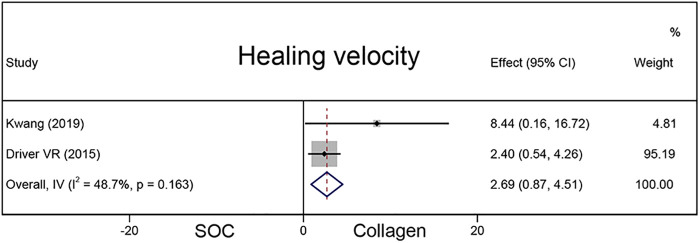
The pooled result of healing velocity.

A total of 337 chronic wounds in two studies were included to compare the healing velocity between the collagen and SOC groups (Figure 5). Compared with the SOC group, the collagen group had a higher average wound area reduction (MD = 2.69; 95% CI, 0.87–4.51).

#### Recurrence of ulceration

**Figure 6:**
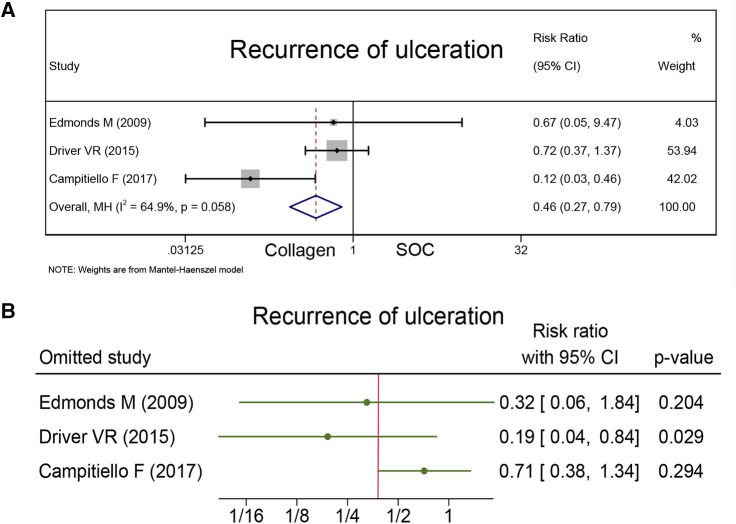
Recurrence of ulceration: (**A**) the pooled result; (**B**) sensitivity analysis.

The recurrence of ulceration was reported as a safety outcome in three studies together with 172 healing wounds. Campitiello et al. indicated that the number of wounds healed in the collagen and control groups during the 6-week observation period was 20 and 12, respectively, and the rate of recurrence was 8.69% for the collagen group and 43.47% for the SOC group. Driver et al. and Edmonds et al. indicated that the rate of recurrence was 19% for the collagen group and 26% for the SOC group (*P* = 0.32), and 7% for the collagen group and 10% for the SOC group during 12-week treatment (*P* = 1.00). As Figure 6A presents that the collagen group had a less recurrence rate than the control group (RR = 0.46; 95% CI, 0.27–0.79).

#### Adverse events

**Figure 7:**
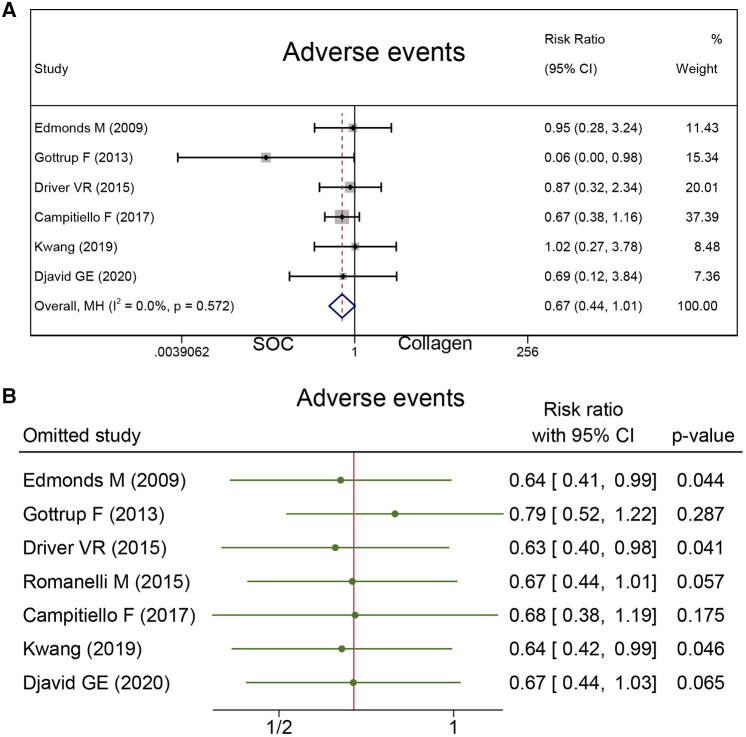
Adverse events: (**A**) the pooled result; (**B**) sensitivity analysis.

To measure the safety of collagen dressing, adverse events were reported in seven studies with 556 cases. There was no difference in the risk of adverse events between the collagen group and SOC group (RR = 0.67; 95% CI, 0.44–1.01), as presented in Figure 7A.

### Sensitivity analysis and GRADE assessment

Sensitivity analysis was conducted, and the results are presented in Figures 4B, 6B, and 7B. The sensitivity analysis demonstrated that the results were stable. Table 4 shows the overall evidence of each outcome. The certainty level of wound healing rate and recurrence of ulceration was moderate, meanwhile, the evidence level of healing velocity and adverse events were high.

**Table 4:** GRADE assessment for outcomes reported in RCTs on collagen vs. standard of care for chronic wounds.

Outcomes	No. of studies	Study design	Risk of bias	Inconsistency	Indirectness	Imprecision	Other considerations	No. of patients		Relative effect (95% CI)	Certainty^a^
								Collagen	SOC		
Wound healing rate	11	RCT	Serious^b^	No	No	No	No	484	473	RR = 1.53 (1.33, 1.77)	⊕⊕⊕○ Moderate
Healing velocity	2	RCT	No	No	No	No	No	171	166	MD = 2.69 (0.87, 4.51)	⊕⊕⊕⊕ High
Recurrence of ulceration	3	RCT	No	Serious^c^	No	No	No	114	53	RR = 0.47 (0.27, 0.79)	⊕⊕⊕○ Moderate
Adverse events	7	RCT	No	No	No	No	No	281	274	RR = 0.67 (0.44, 1.01)	⊕⊕⊕⊕ High

RCT, randomized controlled trial; MD, mean difference; CI, confidence interval; RR, risk ratio; SOC, standard of care.

a GRADE Working Group grades of evidence: High quality = we are very confident that the true effect lies close to that of the estimate of the effect.; Moderate quality = we are moderately confident in the effect estimate: the true effect is likely to be close to the estimate of the effect, but there is a possibility that it is substantially different; Low quality = our confidence in the effect estimate is limited: the true effect may be substantially different from the estimate of the effect; Very low quality = we have very little confidence in the effect estimate: the true effect is likely to be substantially different from the estimate of effect.

b Downgraded one level for concerns with performance bias.

c Downgraded one level for *I*^2^ > 50%.

### TSA results

**Figure 8:**
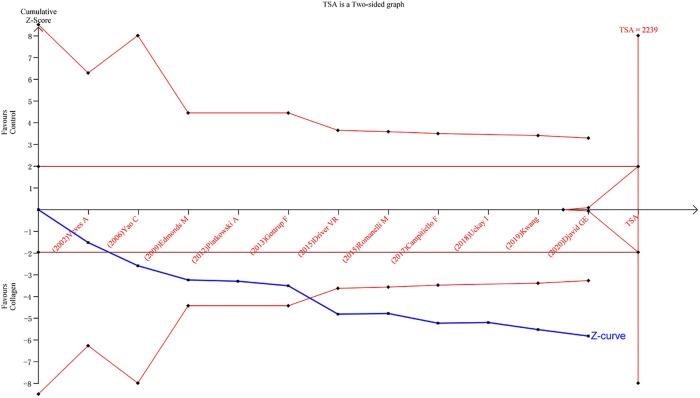
Results of TSA with the primary outcome. The required information size was calculated based on a two-side *α* = 5%, power 80%, and a relative risk reduction of 20%.

TSA was carried out to reduce the risk of Type I error and to evaluate the RIS by keeping the overall 5% risk of Type I error and the relative risk reduction of 20% (power of 80%). For the primary outcome, the result suggested that the *Z*-curve crossed the TSA boundary, and the positive conclusions were obtained in advance, which was consistent with the above meta-analysis results. Hence, it can be asserted that collagen dressing was more effective in the treatment of chronic wounds, and the result was reliable (Figure 8).

## Discussion

This is the first meta-analysis that reviews the efficacy of collagen dressing on chronic wounds. In this article, available studies were combined, and a suitable methodology was used to analyze the clinical value of collagen dressing in the treatment of chronic wounds. The study design was mainly about RCTs, which enhanced the credibility of the results of this article. We specifically focused on four outcomes: wound healing rate, healing velocity, recurrence of ulceration, and adverse events. At the same time, subgroup analysis was also performed according to follow-up and wound types. In the current study, we found collagen dressing had an excellent effect on chronic wounds, which facilitated wound closure. At the same time, adverse events were similar in both groups and no immunological rejection was reported in those studies. Infections were recorded in the collagen group (8.3%) and the control group (15.0%), which suggested collagen dressing was safe and reliable.

The cost of mass production of dressings from human collagen is very high. In practice, recombinant human collagen (28) or animal collagen is used as raw materials. Most of the animal collagens are the Achilles tendon of horses and bovines or the skin of pigs or bovines (29). The process of collagen extraction is complex, a series of special gentle extraction and purification processes are applied to ensure the stability of the original collagen structure, reduce foreign body reactions and increase purity (30–32). Collagen dressings can be classified by the raw materials used in the production process, production method, and additional ingredients added to the collagen dressing (29). For example, the addition of oxidized regenerated cellulose (ORC) is effective in enhancing the management of wound exudate, while binding free radicals and metal ions to reduce protease levels (33, 34).

Collagen dressings are extremely advantageous in wound healing, which include the following: (1) it has low immune rejection in humans, which is based on the strongly preserved primary sequence and the helix structure (35, 36); (2) collagen binds platelets to trigger a coagulation cascade (37, 38) and is considered a mechanical haemostatic agent because it can slow blood flow and provide a clot-forming matrix (39); (3) due to the special three-dimensional structure of collagen, collagen dressings can absorb fluids many times their own weight, resulting in gelation and creating a moist wound environment, which is beneficial to dressing change; (4) as a competitive substrate for collagenase, it can reduce enzymatic degradation of tissues (40); (5) low pH treated collagen dressings reduce peri-wound pH, thus mitigating the risk of bacterial infection and inhibiting protease activity (41–43); (6) collagen breakdown products reduce the activity of type I collagenase (44); (7) the scaffold structure of collagen dressings chemotactic fibroblasts, macrophages and epithelial cells participates in wound healing (29); and (8) the collagen in the dressing promotes collagen deposition and angiogenesis for faster wound healing (45). A moist wound environment is also beneficial for dressing change, increases the healing rate, and improves the patient's quality of life (21). Due to the abovementioned advantages in wound management, collagen is also applied in skin substitutes (46). Apligraft is the first bio-engineered skin that was approved by FDA in 1998, which is composed of bovine collagen gel and neonatal keratinocytes (47). The successful application of Apligraft in wound management, such as DFUs, VLUs, acute wounds, and epidermolysis bullosa wounds was demonstrated (48, 49). Other skin substitutes that contribute to enhancing wound healing, such as Matriderm, Biobrane, Integra Dermal Regeneration Template, Nevelia, and OrCel also showed excellent clinical effects (46).

Although collagen dressings are costly in short term, the cost of collagen dressings can be offset by improved wound healing rate and accelerated wound healing (50, 51). Accelerating wound healing can be interpreted as reducing society's financial expenditure, as it can reduce the likelihood of incapacity. Simultaneously, it also cuts costs by declining the frequency of dressing changes (52). A previous study demonstrated adding a collagen-containing dressing for treatment DFU management could reduce cost by 22% (from £2,897 to £2,255 per patient) compared with standard care alone (53). Another study on VLU also held the same point of view on this, which reduced the cost of management by 40% (from £6,328 to £3,789 per patient) and improved quality of life (from 0.331 to 0.373 QALYs per patient) (54).

Chronic wound healing is a challenging problem. Tiago et al. (6) demonstrated that collagen has a positive effect on wound healing by reviewing 16 studies (14 studies for animals and only 2 studies for humans). However, a meta-analysis was not performed due to high heterogeneity and patients with diabetes mellitus were excluded whereas we performed a meta-analysis and included diabetes mellitus patients. Apart from typical wounds, collagen dressing is also successfully applied in atypical ulcers. Atypical ulcers, such as hydroxyurea-induced ulcers, ulcers caused by autoimmune diseases, and sickle cell anemia, were regarded as difficult to heal. In a retrospective cohort, Garwood et al. (55) found 30 of 71 atypical ulcers healing after applying bovine collagen matrix, which demonstrated that bovine collagen matrix may a successful treatment for atypical ulcers.

The current limitations of this study should be noted. First, only 11 original studies were included and 2 of them were reported as high risk. Second, the TSA analysis indicated that the number of cases included in this article was inadequate. Third, it also should be noted that the types of collagen dressings used in the included studies were inconsistent, which may contribute to heterogeneity and bias, although we tried to harmonies the dressing criteria as much as possible during the screening phase. Apart from inconsistencies in the type of dressing, there are also inconsistencies in the factors that can influence the results, including the method of measuring wound area and duration time. Fourth, immunological rejection was an issue that requested special concern. Unfortunately, limited by available data, the current systematic not pooled this endpoint. Therefore, future studies need to take these factors into account and more multicenter, randomized controlled clinical studies are needed to further demonstrate those views.

## Conclusion

Collagen dressing combining SOC increases the rate of chronic wound healing, accelerates non-healing wound closure, and reduces ulcer recurrence compared with SOC alone. Therefore, adding extra collagen may be an effective potential wound management that holds the ability to facilitate chronic healing. However, more multicenter, randomized controlled clinical studies are needed to substantiate those points in the future.

## Data Availability

The original contributions presented in the study are included in the article/**Supplementary Material**, further inquiries can be directed to the corresponding author/s.
